# Current immunosuppressive treatment and self-reported lifetime stroke history among United States adults: A cross-sectional study

**DOI:** 10.1371/journal.pone.0355164

**Published:** 2026-07-30

**Authors:** Muhammad Saad Ansari, Fiza Nisar, Ufaq Reyaz, Mahnoor Fatima, Umair Ahmad Khan, Baseerullah Hadi, Muhammad Talha Khalid

**Affiliations:** 1 Neurology, Punjab Medical College, Faisalabad, Pakistan; 2 Neurology, Louisiana State University, Shreveport, United States of America; 3 Faculty of Medicine, Rokhan Institute of Higher Education, Jalalabad, Afghanistan; 4 Internal Medicine, Allied Hospital, Faisalabad, Pakistan; University of Diyala College of Medicine, IRAQ

## Abstract

Current immunosuppressive treatment is used for autoimmune, inflammatory, malignant, transplant-related, and other chronic conditions that may carry elevated cerebrovascular burden. Nationally representative evidence on current immunosuppressive treatment status and self-reported lifetime stroke history remains limited. This cross-sectional study used pooled 2023–2024 National Health Interview Survey Adult Sample data. The analytic sample included adults aged 18 years or older with valid survey weights and complete data on exposure, outcome, and selected covariates. The exposure was current self-reported use of medications that weaken the immune system. The outcome was self-reported lifetime stroke history. Survey-weighted logistic regression estimated odds ratios and 95% confidence intervals, accounting for National Health Interview Survey stratification, clustering, and adjusted sampling weights. The final analytic sample included 60,026 adults, representing approximately 250 million United States adults. The weighted prevalence of current immunosuppressive treatment was 4.6%. Stroke history was more common among adults receiving immunosuppressive treatment than among those not receiving treatment (6.0% versus 2.8%). In unadjusted analysis, immunosuppressive treatment was associated with higher odds of stroke history (odds ratio 2.24, 95% confidence interval 1.86–2.70, p < 0.001). After full adjustment for age, sex, race/ethnicity, hypertension, diabetes, smoking status, coronary heart disease, and income-to-poverty category, the association persisted (adjusted odds ratio 1.71, 95% confidence interval 1.40–2.08, p < 0.001). Current immunosuppressive treatment status, likely reflecting both medication exposure and underlying disease burden, was associated with higher odds of self-reported lifetime stroke history among United States adults. Because of the cross-sectional design, broad exposure definition, self-reported outcome, and potential confounding by indication, these findings should be interpreted as an association rather than evidence of causation.

## Introduction

Stroke is a major cause of morbidity, disability, and mortality in the United States and remains a central focus of cardiovascular and neurologic prevention efforts [1]. Identifying populations with elevated cerebrovascular disease burden is important for risk-factor recognition and public health surveillance.

Adults receiving immunosuppressive treatment may represent one such population. Immunosuppressive medications are used for heterogeneous indications, including systemic autoimmune disease, inflammatory disorders, malignancy-associated treatment, and transplant-related care. These indications frequently overlap with chronic inflammation, immune dysregulation, high comorbidity burden, and intensive health-care utilization, processes that have independently been linked to cerebrovascular risk [2,4]. Nationally representative studies therefore need to distinguish carefully between medication exposure and the underlying disease burden that leads to treatment.

The biologic plausibility linking immune-mediated disease and cerebrovascular disease is supported by literature on inflammation, atherosclerosis, and immune dysregulation [2–5]. Large population-based evidence has also shown that autoimmune diseases are associated with increased risk across multiple cardiovascular outcomes, including cerebrovascular disease [6]. Disease-specific literature has reported elevated cardiovascular or cerebrovascular risk in rheumatoid arthritis, systemic lupus erythematosus, and inflammatory bowel disease [7–10]. Medication-specific observational studies in autoimmune populations suggest that cardiovascular risk may differ by drug class, disease severity, and treatment indication [11–14].

Despite this background, nationally representative United States evidence on the association between current immunosuppressive treatment status and self-reported lifetime stroke history is limited. This study used pooled 2023–2024 National Health Interview Survey Adult Sample data to examine whether current self-reported immunosuppressive treatment was associated with self-reported lifetime stroke history among United States adults. We hypothesized that adults reporting current immunosuppressive treatment would have higher odds of reporting prior stroke after adjustment for demographic, socioeconomic, behavioral, and cardiometabolic factors. Because the National Health Interview Survey ascertains stroke history through a single self-reported item without physician confirmation or subtype classification, “stroke” in this study refers broadly to any self-reported lifetime cerebrovascular event and does not distinguish ischemic from hemorrhagic subtype.

## Materials and methods

### Study design and data source

This cross-sectional study used pooled 2023–2024 National Health Interview Survey Adult Sample data. The National Health Interview Survey is a nationally representative, stratified, multistage probability survey of the civilian, noninstitutionalized United States population conducted by the National Center for Health Statistics [15]. National Health Interview Survey sampling weights, strata, and primary sampling units are designed to support population-representative estimation and valid variance estimation when the complex survey design is incorporated [15]. The Strengthening the Reporting of Observational Studies in Epidemiology guidance was used to structure reporting [16].

The 2023 and 2024 public-use data files were accessed for research purposes on 31 May 2026. The authors accessed only de-identified public-use data and had no access to information that could identify individual participants during or after data collection.

### Study population

**Fig 1:**
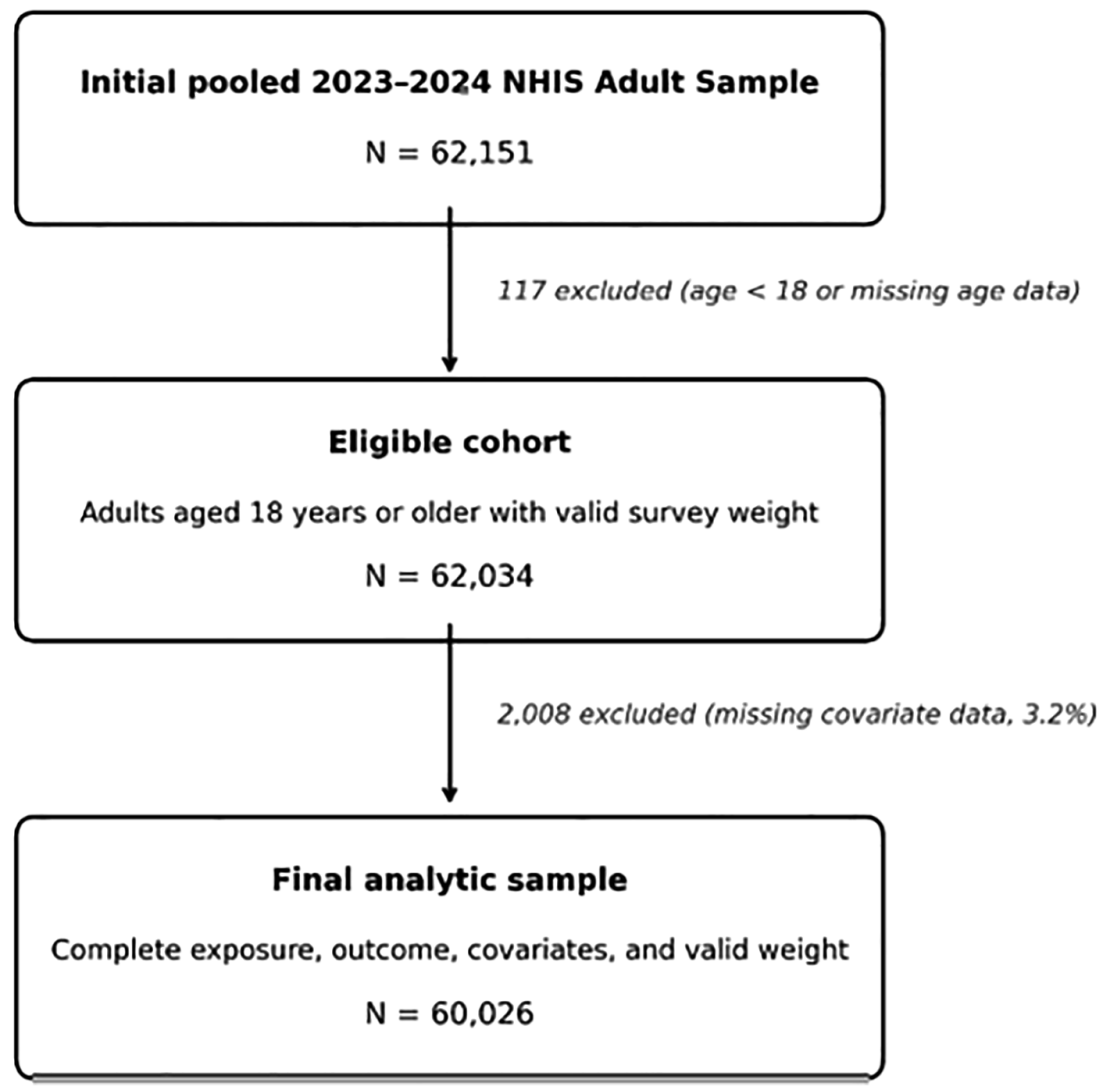
Flow diagram of analytic sample selection from pooled 2023–2024 National Health Interview Survey Adult Sample data. NHIS, National Health Interview Survey.

The study population included adults aged 18 years or older. Participants were eligible if they had valid information on immunosuppressive treatment status and self-reported lifetime stroke history, a positive adult sample weight, and complete data for selected covariates. Survey weights were divided by two to account for pooling two survey years. The final complete-case analytic sample included 60,026 adults. A complete-case analysis was used because the primary regression models required nonmissing data for exposure, outcome, survey design variables, and all selected covariates. Of 62,034 eligible adults, 2,008 were excluded because of missing covariate data, leaving a final analytic sample of 60,026 adults. This represented approximately 3.2% of the eligible sample. Complete-case analysis was considered acceptable because the proportion excluded was small; however, possible selection bias from missing data was acknowledged as a limitation. Fig 1 summarizes sample selection.

### Exposure

The exposure was current self-reported immunosuppressive treatment status, based on the National Health Interview Survey item regarding current use of medications that weaken the immune system (MEDRXTRT_A). The primary exposure was coded as yes versus no. The National Health Interview Survey immunosuppressive treatment item does not capture medication class, dose, treatment indication, or disease severity; therefore, medication-specific or indication-specific associations with stroke could not be examined, and the exposure should be interpreted as a broad marker of immunosuppressive treatment status rather than a specific therapeutic agent. Refused or “don’t know” responses for current immunosuppressive treatment were uncommon, occurring in 186 of 62,034 eligible participants (0.30%; refused: n = 36; don’t know: n = 150). These responses were grouped with the no-treatment category in the primary binary analysis to preserve a conservative reference group and avoid unstable estimates from sparse categories; a sensitivity analysis modeled uncertain responses separately to evaluate whether this coding decision affected the findings.

### Outcome

The primary outcome was self-reported lifetime stroke history, defined using STREV_A. Participants reporting a prior stroke were classified as having stroke history. The analysis estimates the association between current treatment status and lifetime stroke history, not incident stroke or prospective stroke risk.

### Covariates

Covariates were selected a priori based on known or suspected associations with both immunosuppressive treatment status and stroke history, and because they are consistently ascertained and validated within the National Health Interview Survey Adult Sample core module. These included age, sex, race/ethnicity, hypertension, type 2 diabetes, smoking status, coronary heart disease, and income-to-poverty category. Other established stroke-related factors, including body mass index, hyperlipidemia, physical activity level, atrial fibrillation, and chronic kidney disease, were not included in the primary adjustment set; their omission is addressed as a source of potential residual confounding in the Limitations. Race/ethnicity was categorized as non-Hispanic White, non-Hispanic Black, Hispanic, or Other. Smoking status was categorized as never smoker, current every day smoker, current some day smoker, or former smoker. Income was modeled as an ordinal income-to-poverty category, with higher values representing higher family income relative to the poverty threshold.

### Statistical analysis

All analyses incorporated the National Health Interview Survey complex survey design, including adjusted adult sample weights, strata, and primary sampling units. Descriptive statistics were calculated as survey-weighted proportions for categorical variables and survey-weighted means with standard errors for continuous variables. Group comparisons used survey-weighted Wald tests for continuous variables and Rao-Scott design-adjusted chi-square tests for categorical variables. Corresponding survey-weighted test statistics were reported for Table 1.

**Table 1:** Weighted baseline characteristics by current immunosuppressive treatment status.

Characteristic	No treatment	Current treatment	Test statistic	p-value
Unweighted N	57,051	2,975		
Weighted N	238,543,736	11,523,186		
Weighted prevalence	95.4%	4.6%		
Stroke history, weighted %	2.8%	6.0%	Rao-Scott F = 74.73	<0.001
Age, mean (SE), years	47.9 (0.13)	52.4 (0.40)	Wald F = 123.75	<0.001
Female, weighted %	50.9%	59.0%	Rao-Scott F = 46.74	<0.001
Non-Hispanic White, weighted %	61.5%	70.2%	Rao-Scott F = 18.51	<0.001
Non-Hispanic Black, weighted %	11.7%	9.2%		
Hispanic, weighted %	17.9%	13.0%		
Other race/ethnicity, weighted %	8.9%	7.6%		
Hypertension, weighted %	31.5%	42.1%	Rao-Scott F = 116.07	<0.001
Type 2 diabetes, weighted %	9.6%	15.5%	Rao-Scott F = 74.09	<0.001
Coronary heart disease, weighted %	4.7%	9.4%	Rao-Scott F = 84.16	<0.001
Never smoker, weighted %	67.5%	60.4%	Rao-Scott F = 15.38	<0.001
Current every day smoker, weighted %	7.7%	7.8%		
Current some day smoker, weighted %	2.7%	2.8%		
Former smoker, weighted %	22.1%	28.9%		
Income-to-poverty category, mean (SE)	9.78 (0.04)	9.52 (0.10)	Wald F = 6.85	0.009

SE, standard error. All percentages are survey-weighted. Continuous variables were compared using survey-weighted Wald tests. Categorical variables were compared using Rao-Scott design-adjusted chi-square tests. All analyses accounted for National Health Interview Survey stratification, clustering, and sampling weights.

Survey-weighted logistic regression was used to estimate the association between immunosuppressive treatment status and self-reported lifetime stroke history. Logistic regression is appropriate for binary outcomes in cross-sectional data when interpreted as an odds ratio rather than a prospective risk ratio [17]. An unadjusted model included immunosuppressive treatment only. Sequential models then adjusted for potential confounders: Model 1 included age, sex, and race/ethnicity; Model 2 additionally included hypertension, diabetes, and smoking status; and Model 3 additionally included coronary heart disease and income-to-poverty category. Model 3 was considered the primary fully adjusted model. Exploratory interaction analyses examined whether the association differed by sex, age group, race/ethnicity, diabetes status, or hypertension status. No correction was applied for multiple comparisons in exploratory interaction analyses; therefore, subgroup findings were interpreted cautiously. Statistical significance was defined as two-sided p < 0.05. Data extraction, cleaning, and variable transformation were performed using the Lumono.ai research platform; statistical analyses were conducted in R version 4.5 using survey-weighted methods [18].

## Results

### Study population and exposure prevalence

The final analytic sample included 60,026 adults, representing approximately 250 million United States adults after application of survey weights. Overall, 2,975 participants reported current immunosuppressive treatment, corresponding to a weighted prevalence of 4.6%.

### Baseline characteristics

Participants receiving immunosuppressive treatment were older than those not receiving treatment (weighted mean age 52.4 versus 47.9 years). The weighted prevalence of current immunosuppressive treatment was 5.3% among females and 3.9% among males. Adults receiving immunosuppressive treatment had higher comorbidity burden, including hypertension (42.1% versus 31.5%), diabetes (15.5% versus 9.6%), and coronary heart disease (9.4% versus 4.7%). Stroke history was also more common among adults receiving immunosuppressive treatment than among those not receiving treatment (6.0% versus 2.8%, p < 0.001). Baseline characteristics are shown in Table 1.

### Sequential regression models

In the unadjusted survey-weighted logistic regression model, current immunosuppressive treatment was associated with higher odds of self-reported lifetime stroke history (odds ratio 2.24, 95% confidence interval 1.86–2.70, p < 0.001). After adjustment for age, sex, and race/ethnicity, the association was attenuated but remained statistically significant (odds ratio 2.00, 95% confidence interval 1.65–2.44, p < 0.001). Further adjustment for hypertension, diabetes, and smoking status yielded an odds ratio of 1.84 (95% confidence interval 1.51–2.24, p < 0.001). In the fully adjusted model, which additionally included coronary heart disease and income-to-poverty category, current immunosuppressive treatment remained associated with higher odds of stroke history (adjusted odds ratio 1.71, 95% confidence interval 1.40–2.08, p < 0.001). Sequential models are shown in Table 2.

**Table 2:** Sequential survey-weighted logistic regression models for the association between current immunosuppressive treatment and self-reported lifetime stroke history.

Model	Adjustments	OR	95% CI	p-value
Unadjusted	None	2.24	1.86–2.70	<0.001
Model 1	Age, sex, race/ethnicity	2.00	1.65–2.44	<0.001
Model 2	Model 1 + hypertension, diabetes, smoking status	1.84	1.51–2.24	<0.001
Model 3	Model 2 + coronary heart disease, income-to-poverty category	1.71	1.40–2.08	<0.001

CI, confidence interval; OR, odds ratio. All estimates account for the National Health Interview Survey complex survey design.

### Fully adjusted model

In the fully adjusted model, older age, hypertension, diabetes, current smoking, former smoking, coronary heart disease, and lower income-to-poverty category were associated with higher odds of stroke history. Coronary heart disease (odds ratio 2.41, 95% confidence interval 2.10–2.78) and hypertension (odds ratio 2.25, 95% confidence interval 1.95–2.59) showed strong independent associations with stroke history. Full model estimates are shown in Table 3.

**Table 3:** Fully adjusted survey-weighted logistic regression model for self-reported lifetime stroke history.

Variable	OR	95% CI	p-value
Current immunosuppressive treatment	1.71	1.40–2.08	<0.001
Age, per year	1.04	1.04–1.05	<0.001
Female vs male	0.91	0.80–1.02	0.100
Non-Hispanic Black vs non-Hispanic White	1.21	1.02–1.44	0.030
Hispanic vs non-Hispanic White	0.78	0.65–0.94	0.009
Other vs non-Hispanic White	0.97	0.77–1.21	0.758
Hypertension	2.25	1.95–2.59	<0.001
Type 2 diabetes	1.42	1.25–1.62	<0.001
Current every day smoker vs never	1.64	1.37–1.97	<0.001
Current some day smoker vs never	1.63	1.22–2.18	0.001
Former smoker vs never	1.25	1.11–1.40	<0.001
Coronary heart disease	2.41	2.10–2.78	<0.001
Income-to-poverty category	0.91	0.90–0.92	<0.001

CI, confidence interval; OR, odds ratio. Model includes all variables shown and accounts for the National Health Interview Survey complex survey design.

### Exploratory interaction and sensitivity analyses

Exploratory interaction analyses suggested possible effect modification by age and race/ethnicity. The association between immunosuppressive treatment and stroke history was stronger among adults aged 55 years or younger (odds ratio 2.67, 95% confidence interval 1.76–4.06) than among adults older than 55 years (odds ratio 1.35, 95% confidence interval 1.10–1.67; interaction p = 0.018). Evidence of possible interaction was also observed by race/ethnicity (interaction p = 0.041). No statistically significant interaction was observed by sex, diabetes status, or hypertension status. Because multiple subgroup tests were performed and no correction was applied for multiple testing, these findings should be interpreted as exploratory.

In sensitivity analysis using a three-level exposure definition, the association between definite immunosuppressive treatment and stroke history was nearly identical to the primary analysis (odds ratio 1.71, 95% confidence interval 1.41–2.09, p < 0.001). Participants with uncertain immunosuppressive treatment status also had higher odds of stroke history than those reporting no immunosuppressive treatment (odds ratio 2.03, 95% confidence interval 1.03–4.02, p = 0.041). Sensitivity analysis results are shown in Table 4. A supplementary analysis addressed outcome-ascertainment uncertainty specifically: of the 60,026 participants in the analytic sample, 22 (0.04%) had a refused or “don’t know” response for stroke history, coded as no stroke history in the primary analysis. Excluding these 22 participants did not change the fully adjusted association (odds ratio 1.71, 95% confidence interval 1.40–2.08), and recoding them as having stroke history, a conservative worst-case assumption, attenuated the association only slightly (odds ratio 1.69, 95% confidence interval 1.39–2.06). These findings indicate that the primary analysis’s treatment of uncertain stroke-history responses did not materially affect the conclusions. A related sensitivity analysis added the two of these factors that are measurable in the National Health Interview Survey, body mass index and hyperlipidemia, to the fully adjusted model. The association was essentially unchanged (odds ratio 1.72, 95% confidence interval 1.41–2.09, versus 1.71 in the primary model), indicating that these two factors do not materially confound the association. Physical activity and atrial fibrillation could not be assessed because the National Health Interview Survey does not collect these variables.

**Table 4:** Sensitivity analysis using a three-level immunosuppressive treatment exposure definition.

Analysis	Comparison	OR	95% CI	p-value
Primary binary exposure	Yes vs no/uncertain	1.71	1.40–2.08	<0.001
Three-level exposure	Yes vs no	1.71	1.41–2.09	<0.001
Three-level exposure	Uncertain vs no	2.03	1.03–4.02	0.041

CI, confidence interval; OR, odds ratio. All models used the same adjustment set as the fully adjusted primary model.

## Discussion

### Principal findings

In this nationally representative cross-sectional study of United States adults, current self-reported immunosuppressive treatment status was associated with higher odds of self-reported lifetime stroke history. Adults receiving immunosuppressive treatment had a higher weighted prevalence of stroke history than adults not receiving treatment. Sequential adjustment attenuated the association, suggesting partial confounding by demographic and cardiometabolic factors, but the association remained statistically significant after full adjustment.

### Interpretation

The findings should be interpreted as evidence that current immunosuppressive treatment status identifies a medically complex population with higher cerebrovascular disease burden. They should not be interpreted as evidence that immunosuppressive medications caused stroke. The exposure likely captures both medication use and the underlying conditions requiring treatment. The observed association is consistent with evidence linking immune-mediated disease, chronic inflammation, and vascular outcomes [2–6]. It is also consistent with disease-specific literature reporting elevated cardiovascular or cerebrovascular risk among patients with rheumatoid arthritis, systemic lupus erythematosus, and inflammatory bowel disease [7–10].

The broad immunosuppressive treatment variable likely captures a heterogeneous group of underlying conditions, including autoimmune and inflammatory disorders, transplant-related immunosuppression, malignancy-associated treatment, and other chronic diseases requiring immune-modifying therapy. These conditions may differ substantially in their vascular risk profiles. For example, systemic autoimmune diseases may increase cerebrovascular burden through chronic inflammation, endothelial dysfunction, thrombosis, and accelerated atherosclerosis, whereas transplant- or malignancy-related immunosuppression may reflect different pathways of medical complexity. Because the National Health Interview Survey does not identify medication class, dose, treatment indication, disease activity, or timing relative to stroke in this analysis, the observed association should be interpreted as reflecting a broad marker of immunosuppressive treatment status and underlying disease burden rather than a medication-specific effect.

Medication-specific studies support caution in interpretation. Observational research among rheumatoid arthritis and systemic lupus erythematosus populations suggests that cardiovascular event risk may vary by medication class, disease severity, and patient selection [11,12]. Studies of autoimmune disease and immune-related therapies further show that cardiovascular outcomes can reflect complex interactions among underlying disease, treatment, and baseline risk profile [13,14].

### Clinical and public health implications

These findings do not support changing immunosuppressive therapy decisions. Rather, they suggest that adults reporting current immunosuppressive treatment may warrant careful attention to conventional stroke-related factors, including hypertension, diabetes, smoking, coronary heart disease, and socioeconomic barriers to care. Clinicians should interpret current immunosuppressive treatment status as a possible marker of medical complexity and elevated cerebrovascular burden, not as a medication-specific causal risk factor.

### Strengths and limitations

Strengths of this study include use of a large nationally representative United States sample, incorporation of National Health Interview Survey weights and design variables, adjustment for major demographic and cardiometabolic confounders, sequential modeling to assess attenuation across adjustment steps, and sensitivity analysis using an alternative exposure definition. The reporting structure followed Strengthening the Reporting of Observational Studies in Epidemiology guidance for observational studies [16].

This study also has important limitations. First, the cross-sectional design prevents assessment of temporality. Current immunosuppressive treatment may have started before or after a participant’s stroke; therefore, this study estimates an association with lifetime stroke history, not incident stroke risk. Second, both exposure and outcome were self-reported, which may introduce misclassification. Third, the exposure was broad and did not distinguish medication class, dose, duration, indication, or disease activity. Fourth, confounding by indication and residual confounding are major concerns because the analysis could not adjust for specific autoimmune diagnoses, cancer history, transplant status, chronic kidney disease, body mass index, hyperlipidemia, physical activity level, atrial fibrillation, disease severity, corticosteroid dose, medication duration, or health-care utilization. Fifth, refused or “don’t know” responses for stroke history were treated as negative in the primary analysis, which may have underestimated stroke prevalence. Sixth, complete-case analysis may introduce selection bias if missing covariate data were related to treatment status or stroke history, although the proportion excluded for missing confounder data was relatively small. Finally, the National Health Interview Survey excludes institutionalized adults, which may underestimate stroke burden among adults with severe disability or long-term care needs.

## Conclusions

In pooled 2023–2024 National Health Interview Survey Adult Sample data, current self-reported immunosuppressive treatment status was associated with higher odds of self-reported lifetime stroke history among United States adults. The association persisted after adjustment for demographic, socioeconomic, behavioral, and cardiometabolic factors. These findings suggest that adults receiving immunosuppressive treatment may represent a population with elevated cerebrovascular disease burden. However, because of the cross-sectional design, broad exposure definition, self-reported outcome, and likely confounding by indication, causality cannot be inferred. Longitudinal studies with medication-specific exposure data, clinical stroke ascertainment, treatment indication, disease severity, and timing of exposure relative to stroke are needed.

## Supporting information

S1 File R code used for statistical analysis. R script implementing all descriptive statistics, regression models, interaction analyses, and sensitivity analyses reported in this manuscript, including the sequential models in Table 2 and the sensitivity analyses in Table 4. Instructions for downloading and pooling the raw National Health Interview Survey files are included in the script.
(R)

S2 File STROBE checklist. Completed Strengthening the Reporting of Observational Studies in Epidemiology (STROBE) checklist for this cross-sectional study, with page references to the manuscript.
(DOCX)
