# Supplementary material for: Current immunosuppressive treatment and self-reported lifetime stroke history among United States adults: A cross-sectional study
Source: PLoS One. 2026 Jul 30;21(7):e0355164. doi: 10.1371/journal.pone.0355164 (PMC13422849; doi:10.1371/journal.pone.0355164)
Supplement: S2 File — Completed Strengthening the Reporting of Observational Studies in Epidemiology (STROBE) checklist for this cross-sectional study, with page references to the manuscript. (DOCX) [file pone.0355164.s002.docx]

**STROBE Statement—Checklist for Cross-Sectional Studies**

*Manuscript: Current immunosuppressive treatment and self-reported lifetime stroke history among United States adults: a cross-sectional study*

Journal submission: PLOS ONE

Page references correspond to the current double-spaced, continuously line-numbered PLOS ONE manuscript. Items not applicable to this cross-sectional secondary analysis are identified explicitly.

| **Section** | **Item** | **Recommendation** | **Reported on page / location** |
| --- | --- | --- | --- |
| **Title and abstract** | **1** | (a) Indicate the study’s design with a commonly used term in the title or abstract. (b) Provide in the abstract an informative and balanced summary of what was done and what was found. | Title: p. 1. Abstract: pp. 2–3. |
| **Introduction** | **2** | Explain the scientific background and rationale for the investigation being reported. | Introduction, pp. 3–4. |
| **Introduction** | **3** | State specific objectives, including any prespecified hypotheses. | Final paragraph of Introduction, p. 4. |
| **Methods** | **4** | Present key elements of study design early in the paper. | Study design and data source, p. 4. |
| **Methods** | **5** | Describe the setting, locations, and relevant dates, including periods of recruitment, exposure, follow-up, and data collection. | Study design and data source, p. 4: pooled 2023–2024 NHIS Adult Sample; U.S. civilian, noninstitutionalized population. No follow-up because the study is cross-sectional. |
| **Methods** | **6(a)** | Give the eligibility criteria and the sources and methods of selection of participants. | Study population, pp. 4–5; Fig 1 caption, p. 4. |
| **Methods** | **6(b)** | For matched studies, give matching criteria and numbers of exposed and unexposed. | Not applicable; this was not a matched study. |
| **Methods** | **7** | Clearly define all outcomes, exposures, predictors, potential confounders, and effect modifiers. Give diagnostic criteria, if applicable. | Exposure, p. 5; Outcome and Covariates, pp. 5–6; interaction variables, pp. 6–7. |
| **Methods** | **8** | For each variable of interest, give sources of data and details of methods of assessment. Describe comparability of assessment methods if there is more than one group. | Study design and data source, p. 4; Exposure, Outcome, and Covariates, pp. 5–6. All variables were obtained from the same NHIS Adult Sample instrument. |
| **Methods** | **9** | Describe any efforts to address potential sources of bias. | Study population and exposure coding, p. 5; Statistical analysis, pp. 6–7; Strengths and limitations, pp. 13–14. Complete-case analysis, survey weighting, adjusted models, interaction analyses, and sensitivity analysis are described. |
| **Methods** | **10** | Explain how the study size was arrived at. | Study population, pp. 4–5. All eligible respondents in pooled 2023–2024 NHIS data were included; no a priori power calculation was used. |
| **Methods** | **11** | Explain how quantitative variables were handled in the analyses. If applicable, describe which groupings were chosen and why. | Covariates, p. 6; Statistical analysis, pp. 6–7. Age was modeled per year and income as an ordinal income-to-poverty category; categorical coding is described. |
| **Methods** | **12(a)** | Describe all statistical methods, including those used to control for confounding. | Statistical analysis, pp. 6–7. |
| **Methods** | **12(b)** | Describe any methods used to examine subgroups and interactions. | Statistical analysis, p. 7; exploratory interaction analyses, pp. 10–11. |
| **Methods** | **12(c)** | Explain how missing data were addressed. | Study population, p. 5; complete-case analysis and excluded counts are reported. Limitations, pp. 13–14. |
| **Methods** | **12(d)** | If applicable, describe analytical methods taking account of sampling strategy. | Statistical analysis, pp. 6–7: adjusted survey weights, strata, primary sampling units, and survey-weighted procedures. |
| **Methods** | **12(e)** | Describe any sensitivity analyses. | Statistical analysis, p. 7; sensitivity analysis results, p. 11 and Table 4. |
| **Results** | **13(a)** | Report numbers of individuals at each stage of the study—for example, potentially eligible, examined for eligibility, confirmed eligible, included, and analysed. | Study population, p. 5; Fig 1 caption, p. 4. Counts: 62,151 extracted, 62,034 eligible, 60,026 analysed. |
| **Results** | **13(b)** | Give reasons for non-participation at each stage. | Study population, p. 5; Fig 1 caption, p. 4: 117 outside population restriction; 2,008 excluded for missing covariate data. |
| **Results** | **13(c)** | Consider use of a flow diagram. | Fig 1 is supplied as a separate figure file; caption appears on p. 4. |
| **Results** | **14(a)** | Give characteristics of study participants and information on exposures and potential confounders. | Baseline characteristics, pp. 7–8; Table 1, p. 8. |
| **Results** | **14(b)** | Indicate the number of participants with missing data for each variable of interest. | Study population, p. 5, reports 2,008 excluded for missing covariate data. Exposure uncertainty counts are also reported on p. 5. |
| **Results** | **14(c)** | Summarize follow-up time. | Not applicable; this was a cross-sectional study with no follow-up. |
| **Results** | **15** | Report numbers of outcome events or summary measures. | Study population and baseline characteristics, pp. 7–8; Table 1, p. 8: weighted stroke prevalence 6.0% vs 2.8%. |
| **Results** | **16(a)** | Give unadjusted estimates and, if applicable, confounder-adjusted estimates and their precision. Make clear which confounders were adjusted for and why. | Sequential regression models, pp. 9–10; Tables 2 and 3, pp. 9–10. |
| **Results** | **16(b)** | Report category boundaries when continuous variables were categorized. | Covariates, p. 6; age subgroup boundary (≤55 vs >55 years) is reported in interaction results, pp. 10–11. Income category coding is described as ordinal. |
| **Results** | **16(c)** | If relevant, consider translating estimates of relative risk into absolute risk for a meaningful time period. | Not applicable: the study was cross-sectional and reported odds ratios for lifetime stroke history, not prospective risk over time. |
| **Results** | **17** | Report other analyses done—for example, subgroup analyses, interactions, and sensitivity analyses. | Exploratory interaction and sensitivity analyses, pp. 10–11; Table 4, p. 11. |
| **Discussion** | **18** | Summarize key results with reference to study objectives. | Principal findings, pp. 11–12. |
| **Discussion** | **19** | Discuss limitations, taking into account sources of potential bias or imprecision and the direction and magnitude of any potential bias. | Strengths and limitations, pp. 13–14. |
| **Discussion** | **20** | Give a cautious overall interpretation of results considering objectives, limitations, multiplicity of analyses, results from similar studies, and other relevant evidence. | Interpretation and comparison with prior evidence, pp. 12–13; Conclusions, p. 14. |
| **Discussion** | **21** | Discuss the generalisability (external validity) of the study results. | Strengths and limitations, pp. 13–14: nationally representative civilian, noninstitutionalized U.S. adults; exclusion of institutionalized adults is noted. |
| **Other information** | **22** | Give the source of funding and the role of the funders for the present study and, if applicable, for the original study on which the present article is based. | Financial disclosure is entered in the PLOS ONE submission system, as required by the journal. No external funding was received. |
