# Supplementary material for: Current immunosuppressive treatment and self-reported lifetime stroke history among United States adults: A cross-sectional study
Source: PLoS One. 2026 Jul 30;21(7):e0355164. doi: 10.1371/journal.pone.0355164 (PMC13422849; doi:10.1371/journal.pone.0355164)
Supplement: Attachment — Submitted filename: Response to Reviewers.docx [file pone.0355164.s004.docx]

**Response to Editor and Reviewer Comments**

*Current Immunosuppressive Treatment and Self-Reported Lifetime Stroke History Among United States Adults: A Cross-Sectional Study*

PLOS ONE submission PONE-S-26-38063

We thank the editor and reviewer for a careful reading of the manuscript. We address each point below and have made corresponding changes in the revised manuscript, tracked so they can be reviewed directly. Table and figure numbers referenced below match the tracked-changes file.

**Editor Comments**

***Comment:*** *Please ensure that your manuscript meets PLOS ONE's style requirements, including those for file naming.*

**Response:** We reviewed the manuscript against the PLOS ONE formatting templates for the main body and for the title, authors, and affiliations page. The reference list already follows PLOS ONE's numbered, Vancouver-based citation style with DOIs. We would appreciate a check at the production stage for any remaining file-naming or formatting details specific to the submission system.

***Comment:*** *If the reviewer comments include a recommendation to cite specific previously published works, please review and evaluate these publications to determine whether they are relevant.*

**Response:** The editor's letter and the reviewer's comments did not name specific published works for us to evaluate, so no citations were added on this basis.

***Comment:*** *There are many paragraphs in the introduction without references. The term “stroke” is broad and necessitates specification.*

**Response:** We added a citation to the previously uncited sentence in the introduction describing the overlap between immunosuppressive treatment indications and chronic inflammation, immune dysregulation, comorbidity burden, and health care utilization. We also added a sentence, placed after the study hypothesis, stating that the National Health Interview Survey ascertains stroke history through a single self-reported item without physician confirmation or subtype classification, so “stroke” in this study refers broadly to any self-reported lifetime cerebrovascular event and does not distinguish ischemic from hemorrhagic subtype.

***Comment:*** *Methods: the term is broad. What are the categories of immunosuppressants? The immunosuppressive agents were prescribed to pathological conditions, which may act as confounding factors.*

**Response:** We added a sentence to the Methods (Exposure subsection) stating directly that the National Health Interview Survey item underlying our exposure, MEDRXTRT_A, is a single yes/no question with no information on medication class, dose, treatment indication, or disease severity, and that medication-specific or indication-specific associations with stroke could not be examined for this reason. We also expanded the Methods (Covariates subsection) to justify why the eight included covariates were selected, and expanded the Limitations to name the specific stroke-related factors we could not adjust for: body mass index, hyperlipidemia, physical activity, atrial fibrillation, chronic kidney disease, specific autoimmune diagnoses, cancer history, transplant status, disease severity, corticosteroid dose, medication duration, and health care utilization.

***Comment:*** *Format the references according to the guidelines.*

**Response:** We audited every reference against PLOS ONE's citation style (numbered, Vancouver-based, first six authors followed by ‘et al.’) and against the source each citation points to, checking titles, author lists, journal, year, volume, pagination, and DOI. This audit found and corrected nine errors. Six references (1, 5, 6, 11, 12, 13) listed only three authors followed by ‘et al.’ where PLOS ONE style requires the first six; we restored the full six-author lists for each. Reference 12 also carried an incorrect DOI that resolved to an unrelated editorial rather than the intended article; we corrected it to the DOI of the article actually cited (Choi MY, et al., Ann Rheum Dis). Reference 14 cited an incorrect year and page range; we corrected it to 2023;113: 246–259, matching the article's actual publication record, and corrected the author list to end at the sixth author with no ‘et al.’, since the article has exactly six authors. A ninth reference (originally attributed to Ungprasert et al., on inflammatory bowel disease and cerebrovascular risk) could not be verified: the DOI given in the manuscript resolved to an unrelated paper, and no matching article could be located in PubMed or by general search under that title or author list. We replaced it with a verified, directly relevant reference supporting the same statement: Xiao Z, Pei Z, Yuan M, Li X, Chen S, Xu L. Risk of stroke in patients with inflammatory bowel disease: a systematic review and meta-analysis. J Stroke Cerebrovasc Dis. 2015;24: 2774-2780. doi: 10.1016/j.jstrokecerebrovasdis.2015.08.008.

**Reviewer #1**

***Comment:*** *Because the study is cross-sectional, it is impossible to determine whether immunosuppressive treatment occurred before or after the stroke. The conclusions should be narrowed to state only that current immunosuppressive treatment status is associated with a higher prevalence of reported prior stroke.*

**Response:** We reviewed the abstract, discussion, and conclusion and confirmed the language throughout already reflects this: the manuscript states that current immunosuppressive treatment status is associated with higher odds of self-reported lifetime stroke history, that this should be interpreted as an association rather than evidence of causation, and explicitly that the findings should not be read as evidence that immunosuppressive medications caused stroke. No further language changes were needed on this point.

***Comment:*** *The exposure definition is also too broad. Without information on medication class, dose, duration, treatment indication, disease severity, corticosteroid exposure, cancer history, transplant status, chronic kidney disease, or health care utilization, confounding by indication remains a major concern.*

**Response:** Addressed above under the corresponding editor comment: we added a direct statement in Methods that the exposure item cannot support medication-class or indication-specific analysis, and expanded the Limitations to name each of the factors the reviewer lists as unmeasured confounders.

***Comment:*** *The adjustment set is limited. Important stroke-related variables such as BMI, hyperlipidemia, physical activity, kidney disease, atrial fibrillation, medication indication, and disease severity were not included. The authors should provide stronger justification for the selected covariates and discuss the likely impact of residual confounding more directly.*

**Response:** We expanded the Methods to justify covariate selection and, rather than only discussing the likely impact of the missing variables, we tested it directly. Of the factors the reviewer names, the National Health Interview Survey collects body mass index and hyperlipidemia; physical activity and atrial fibrillation are not collected in this survey and remain unmeasured. We added body mass index and hyperlipidemia to the fully adjusted model and reran the analysis. The odds ratio for the primary exposure moved from 1.71 (95% confidence interval 1.40–2.08) to 1.72 (95% confidence interval 1.41–2.09), a negligible change. We report this as a new sensitivity analysis in the Results and state plainly in the Limitations that physical activity and atrial fibrillation could not be assessed because the survey does not collect them.

***Comment:*** *The handling of missing and uncertain responses requires more explanation. Treating refused or “don’t know” responses as negative may underestimate the outcome or exposure prevalence. Additional sensitivity analyses excluding uncertain responses would strengthen the analysis.*

**Response:** The manuscript's existing sensitivity analysis (Table 4) already addresses uncertain responses on the exposure side using a three-level exposure definition. For this revision we conducted the corresponding analysis on the outcome side. Of the 60,026 participants in the analytic sample, 22 (0.04%) gave a refused or “don’t know” response for stroke history, which the primary analysis coded as no stroke history. Excluding these 22 participants left the fully adjusted odds ratio unchanged at 1.71 (95% confidence interval 1.40–2.08). Recoding them as having a stroke history, a conservative worst-case assumption, moved the odds ratio only slightly, to 1.69 (95% confidence interval 1.39–2.06). We added a sentence reporting both results in the Results (sensitivity analyses section).

***Comment:*** *The exploratory subgroup and interaction analyses should be clearly described as hypothesis-generating, especially because multiple comparisons were performed without correction.*

**Response:** The manuscript already states that because multiple subgroup tests were performed without correction for multiple comparisons, these findings should be interpreted as exploratory. We reviewed this language and found no further change was needed.

***Comment:*** *The authors should provide the analytic code, variable recoding decisions, weighting procedures, exclusion criteria, and model syntax to allow full reproducibility.*

**Response:** We have prepared the full analytic code, variable recoding logic, survey design and weighting procedure, exclusion criteria, and model syntax as a Supporting Information file (S1 File, S1_analysis_code.R) accompanying the revised manuscript. It includes the original descriptive, univariable, regression, interaction, sequential-model, and robustness code, plus the three additional blocks added for this revision: the exact Table 2 model sequence, the outcome-side refused/don't-know sensitivity analysis, and the BMI/hyperlipidemia sensitivity analysis.

We thank the editor and reviewer again for the time spent on this manuscript and believe the changes described above address the concerns raised.
