# Supplementary material for: Current immunosuppressive treatment and self-reported lifetime stroke history among United States adults: A cross-sectional study
Source: PLoS One. 2026 Jul 30;21(7):e0355164. doi: 10.1371/journal.pone.0355164 (PMC13422849; doi:10.1371/journal.pone.0355164)
Supplement: Peer review history

# Peer review: Decision Letter 0

**DOI:** 10.1371/journal.pone.0355164.r001

**Authors:** Marwan Al-Nimer

3 Jul 2026

PONE-D-26-28760

Current immunosuppressive treatment and self-reported lifetime stroke history among United States adults: a cross-sectional study

PLOS One

Dear Dr. Hadi,

Thank you for submitting your manuscript to PLOS ONE. After careful consideration, we feel that it has merit but does not fully meet PLOS ONE’s publication criteria as it currently stands. Therefore, we invite you to submit a revised version of the manuscript that addresses the points raised during the review process.

**ACADEMIC EDITOR: Major revision**

Please submit your revised manuscript by Sep 01 2026 11:59PM. If you will need more time than this to complete your revisions, please reply to this message or contact the journal office at plosone@plos.org. When you're ready to submit your revision, log on to https://www.editorialmanager.com/pone/ and select the 'Submissions Needing Revision' folder to locate your manuscript file.

Please include the following items when submitting your revised manuscript:

- A letter that responds to each point raised by the academic editor and reviewer(s). You should upload this letter as a separate file labeled 'Response to Reviewers'.
- A marked-up copy of your manuscript that highlights changes made to the original version. You should upload this as a separate file labeled 'Revised Manuscript with Track Changes'.
- An unmarked version of your revised paper without tracked changes. You should upload this as a separate file labeled 'Manuscript'.

If you would like to make changes to your financial disclosure, please include your updated statement in your cover letter. Guidelines for resubmitting your figure files are available below the reviewer comments at the end of this letter.

If applicable, we recommend that you deposit your laboratory protocols in protocols.io to enhance the reproducibility of your results. Protocols.io assigns your protocol its own identifier (DOI) so that it can be cited independently in the future. For instructions see: https://journals.plos.org/plosone/s/submission-guidelines#loc-laboratory-protocols. Additionally, PLOS ONE offers an option for publishing peer-reviewed Lab Protocol articles, which describe protocols hosted on protocols.io. Read more information on sharing protocols at https://plos.org/protocols?utm_medium=editorial-email&utm_source=authorletters&utm_campaign=protocols.

As the corresponding author, your ORCID iD is verified in the submission system and will appear in the published article. PLOS supports the use of ORCID, and we encourage all coauthors to register for an ORCID iD and use it as well. Please encourage your coauthors to verify their ORCID iD within the submission system before final acceptance, as unverified ORCID iDs will not appear in the published article. *Only*  the individual author can complete the verification step; PLOS staff *cannot*  verify ORCID iDs on behalf of authors.

We look forward to receiving your revised manuscript.

Kind regards,

Marwan Salih Al-Nimer, MD, PhD

Academic Editor

PLOS One

Journal Requirements:

When submitting your revision, we need you to address these additional requirements.

1. Please ensure that your manuscript meets PLOS ONE's style requirements, including those for file naming. The PLOS ONE style templates can be found at

https://journals.plos.org/plosone/s/file?id=wjVg/PLOSOne_formatting_sample_main_body.pdf and

https://journals.plos.org/plosone/s/file?id=ba62/PLOSOne_formatting_sample_title_authors_affiliations.pdf

2. If the reviewer comments include a recommendation to cite specific previously published works, please review and evaluate these publications to determine whether they are relevant and should be cited. There is no requirement to cite these works unless the editor has indicated otherwise.

Additional Editor Comments:

The following points need clarification:

1. There are many paragraphs in the introduction without references. The term "stroke" is broad and necessitates specification.

2: Methods: The term is broad. What are the categories of immunosuppressants? The results will identify which immunosuppressive agent is linked with stroke. In addition, the immunosuppressive agents were prescribed to pathological conditions, which may involve as cofounder factors

3: Format the references according to the guidelines.

[Note: HTML markup is below. Please do not edit.]

Reviewers' comments:

Reviewer's Responses to Questions

**Comments to the Author**

1. Is the manuscript technically sound, and do the data support the conclusions?

The manuscript must describe a technically sound piece of scientific research with data that supports the conclusions. Experiments must have been conducted rigorously, with appropriate controls, replication, and sample sizes. The conclusions must be drawn appropriately based on the data presented.

Reviewer #1: Partly

**********

2. Has the statistical analysis been performed appropriately and rigorously?

Reviewer #1: Yes

**********

3. Have the authors made all data underlying the findings in their manuscript fully available?

The PLOS Data policy requires authors to make all data underlying the findings described in their manuscript fully available without restriction, with rare exception (please refer to the Data Availability Statement in the manuscript PDF file). The data should be provided as part of the manuscript or its supporting information, or deposited to a public repository. For example, in addition to summary statistics, the data points behind means, medians and variance measures should be available. If there are restrictions on publicly sharing data—e.g. participant privacy or use of data from a third party—those must be specified.

Reviewer #1: Yes

**********

4. Is the manuscript presented in an intelligible fashion and written in standard English?

PLOS ONE does not copyedit accepted manuscripts, so the language in submitted articles must be clear, correct, and unambiguous. Any typographical or grammatical errors should be corrected at revision, so please note any specific errors here.

Reviewer #1: Yes

**********

5. Review Comments to the Author

Please use the space provided to explain your answers to the questions above. You may also include additional comments for the author, including concerns about dual publication, research ethics, or publication ethics. (Please upload your review as an attachment if it exceeds 20,000 characters)

Reviewer #1: The manuscript addresses an important topic using a large, nationally representative NHIS dataset. The writing is generally clear, and the authors appropriately use survey-weighted analyses. However, several major methodological issues limit the strength and interpretation of the findings.

The main concern is the mismatch between the exposure and outcome. The exposure is current immunosuppressive treatment, while the outcome is self-reported lifetime stroke history. Because the study is cross-sectional, it is impossible to determine whether immunosuppressive treatment occurred before or after the stroke. Therefore, the manuscript cannot support any inference about immunosuppressive treatment as a risk factor for stroke. The conclusions should be narrowed substantially to state only that current immunosuppressive treatment status is associated with a higher prevalence of reported prior stroke.

The exposure definition is also too broad. “Medications that weaken the immune system” may include very different drug classes and clinical indications, such as autoimmune disease, inflammatory disease, malignancy-related treatment, and transplant-related therapy. These groups differ greatly in baseline vascular risk. Without information on medication class, dose, duration, treatment indication, disease severity, corticosteroid exposure, cancer history, transplant status, chronic kidney disease, or health-care utilization, confounding by indication remains a major concern.

The statistical approach is acceptable in principle because the authors used survey-weighted logistic regression and accounted for the NHIS design. However, the adjustment set is limited. Important stroke-related variables such as BMI, hyperlipidemia, physical activity, kidney disease, atrial fibrillation, medication indication, and disease severity were not included. The authors should provide stronger justification for the selected covariates and discuss the likely impact of residual confounding more directly.

The handling of missing and uncertain responses requires more explanation. In particular, treating refused or “don’t know” responses as negative may underestimate the outcome or exposure prevalence. Additional sensitivity analyses excluding uncertain responses would strengthen the analysis. The exploratory subgroup and interaction analyses should also be clearly described as hypothesis-generating, especially because multiple comparisons were performed without correction.

The data availability statement identifies the NHIS public-use dataset, which is appropriate. However, the authors should provide the analytic code, variable recoding decisions, weighting procedures, exclusion criteria, and model syntax to allow full reproducibility.

**********

6. PLOS authors have the option to publish the peer review history of their article (what does this mean?). If published, this will include your full peer review and any attached files.

If you choose “no”, your identity will remain anonymous but your review may still be made public.

**Do you want your identity to be public for this peer review?**  For information about this choice, including consent withdrawal, please see our Privacy Policy.

Reviewer #1: **Yes:** Fahad S. Alshehri

**********

[NOTE: If reviewer comments were submitted as an attachment file, they will be attached to this email and accessible via the submission site. Please log into your account, locate the manuscript record, and check for the action link "View Attachments". If this link does not appear, there are no attachment files.]

To ensure your figures meet our technical requirements, please review our figure guidelines: https://journals.plos.org/plosone/s/figures

You may also use PLOS’s free figure tool, NAAS, to help you prepare publication quality figures: https://journals.plos.org/plosone/s/figures#loc-tools-for-figure-preparation.

NAAS will assess whether your figures meet our technical requirements by comparing each figure against our figure specifications.

# Author comment: Author response to Decision Letter 1

**DOI:** 10.1371/journal.pone.0355164.r002

7 Jul 2026

We thank the Academic Editor and reviewer for their feedback and have addressed each comment point-by-point in the attached "Response to Reviewers" file. Briefly: we tightened the exposure and outcome definitions, added a direct statement on the limits of confounding-by-indication, and ran a new sensitivity analysis adding body mass index and hyperlipidemia to the fully adjusted model. We also ran a sensitivity analysis excluding and recoding refused/don't-know stroke responses, corrected several reference errors found during a full bibliography audit, and are including our full analytic code as a Supporting Information file (S1 File) for reproducibility. All changes are tracked in the revised manuscript.

Attachment Submitted filename: Response to Reviewers.docx

# Editor report: Decision Letter 1

**DOI:** 10.1371/journal.pone.0355164.r003

**Authors:** Marwan Al-Nimer

20 Jul 2026

Current immunosuppressive treatment and self-reported lifetime stroke history among United States adults: a cross-sectional study

PONE-D-26-28760R1

Dear Dr. Baseerullah Hadi,

We’re pleased to inform you that your manuscript has been judged scientifically suitable for publication and will be formally accepted for publication once it meets all outstanding technical requirements.

Within one week, you’ll receive an e-mail detailing the required amendments. When these have been addressed, you’ll receive a formal acceptance letter and your manuscript will be scheduled for publication.

An invoice will be generated when your article is formally accepted. Please note, if your institution has a publishing partnership with PLOS and your article meets the relevant criteria, all or part of your publication costs will be covered. Please make sure your user information is up-to-date by logging into Editorial Manager at Editorial Manager® and clicking the ‘Update My Information' link at the top of the page. For questions related to billing, please contact billing support.

If your institution or institutions have a press office, please notify them about your upcoming paper to help maximize its impact. If they’ll be preparing press materials, please inform our press team as soon as possible -- no later than 48 hours after receiving the formal acceptance. Your manuscript will remain under strict press embargo until 2 pm Eastern Time on the date of publication. For more information, please contact onepress@plos.org.

Kind regards,

Marwan Salih Al-Nimer, MD, PhD

Academic Editor

PLOS One

Additional Editor Comments (optional):

Reviewers' comments:

# Editor report: Acceptance letter

**DOI:** 10.1371/journal.pone.0355164.r004

**Authors:** Marwan Al-Nimer

PONE-D-26-28760R1

PLOS One

Dear Dr. Hadi,

I'm pleased to inform you that your manuscript has been deemed suitable for publication in PLOS One. Congratulations! Your manuscript is now being handed over to our production team.

At this stage, our production department will prepare your paper for publication. This includes ensuring the following:

* All references, tables, and figures are properly cited

* All relevant supporting information is included in the manuscript submission,

* There are no issues that prevent the paper from being properly typeset

You will receive further instructions from the production team, including instructions on how to review your proof when it is ready. Please keep in mind that we are working through a large volume of accepted articles, so please give us a few days to review your paper and let you know the next and final steps.

Lastly, if your institution or institutions have a press office, please let them know about your upcoming paper now to help maximize its impact. If they'll be preparing press materials, please inform our press team within the next 48 hours. Your manuscript will remain under strict press embargo until 2 pm Eastern Time on the date of publication. For more information, please contact onepress@plos.org.

You will receive an invoice from PLOS for your publication fee after your manuscript has reached the completed accept phase. If you receive an email requesting payment before acceptance or for any other service, this may be a phishing scheme. Learn how to identify phishing emails and protect your accounts at https://explore.plos.org/phishing.

If we can help with anything else, please email us at customercare@plos.org.

Thank you for submitting your work to PLOS ONE and supporting open access.

Kind regards,

PLOS ONE Editorial Office Staff

on behalf of

Professor Marwan Salih Al-Nimer

Academic Editor

PLOS One
